# Multiple imputation of multiple multi-item scales when a full imputation model is infeasible

**DOI:** 10.1186/s13104-016-1853-5

**Published:** 2016-01-26

**Authors:** Catrin O. Plumpton, Tim Morris, Dyfrig A. Hughes, Ian R. White

**Affiliations:** Centre for Health Economics and Medicines Evaluation, Bangor University, Ardudwy, Normal Site, Holyhead Road, Bangor, Gwynedd LL57 2PZ UK; MRC Clinical Trials Unit at UCL, Institute of Clinical Trials and Methodology, Aviation House, 125 Kingsway, London, WC2B 6NH UK; London School of Hygiene and Tropical Medicine, Keppel Street, London, WC1E 7HT UK; MRC Biostatistics Unit, Cambridge Institute of Public Health, Robinson Way, Cambridge, CB2 0SR UK

**Keywords:** Missing data, Multiple imputation, Multi-item scale, Survey data

## Abstract

**Background:**

Missing data in a large scale survey presents major challenges. We focus on performing multiple imputation by chained equations when data contain multiple incomplete multi-item scales. Recent authors have proposed imputing such data at the level of the individual item, but this can lead to infeasibly large imputation models.

**Methods:**

We use data gathered from a large multinational survey, where analysis uses separate logistic regression models in each of nine country-specific data sets. In these data, applying multiple imputation by chained equations to the individual scale items is computationally infeasible. We propose an adaptation of multiple imputation by chained equations which imputes the individual scale items but reduces the number of variables in the imputation models by replacing most scale items with scale summary scores. We evaluate the feasibility of the proposed approach and compare it with a complete case analysis. We perform a simulation study to compare the proposed method with alternative approaches: we do this in a simplified setting to allow comparison with the full imputation model.

**Results:**

For the case study, the proposed approach reduces the size of the prediction models from 134 predictors to a maximum of 72 and makes multiple imputation by chained equations computationally feasible. Distributions of imputed data are seen to be consistent with observed data. Results from the regression analysis with multiple imputation are similar to, but more precise than, results for complete case analysis; for the same regression models a 39 % reduction in the standard error is observed. The simulation shows that our proposed method can perform comparably against the alternatives.

**Conclusions:**

By substantially reducing imputation model sizes, our adaptation makes multiple imputation feasible for large scale survey data with multiple multi-item scales. For the data considered, analysis of the multiply imputed data shows greater power and efficiency than complete case analysis. The adaptation of multiple imputation makes better use of available data and can yield substantively different results from simpler techniques.

**Electronic supplementary material:**

The online version of this article (doi:10.1186/s13104-016-1853-5) contains supplementary material, which is available to authorized users.

## Background

Missing data is ubiquitous in research, and survey data is particularly prone to incomplete responses. One problem arising from missing data is a loss of precision and statistical power. However, poor handling of the missing data during analysis can lead to biased results.

When handling missing data, assumptions must be made about the mechanism of missingness; no analysis with missing data is free of such assumptions. Data may be missing completely at random (MCAR), where the probability of missing data is not dependent on either the observed or unobserved data. When data is missing at random (MAR), the probability of the data being missing does not depend upon the unobserved data, but missingness may be related to the observed data. Alternatively, data may be missing not at random (MNAR), whereby missingness is dependent upon the values of the unobserved data, conditional on the observed data [1–3].

Roth, in 1994, stated that despite its importance, conspicuously little research on missing data analysis appeared within the social sciences literature [4]. It is acknowledged that a gap still exists between techniques recommended by methodological literature and those employed in practice; traditional ad-hoc techniques such as deletion and single imputation techniques are still applied routinely [3, 5, 6].

Complete case (CC) analysis is commonly used, and is efficient and valid under MAR, provided missing data occurs only in the outcome. Once missing data occurs in covariates, or in parts of a composite outcome, complete case analyses are inefficient. Also, when the MCAR assumption does not hold, the data is no longer representative of the target population, compromising external generalisability [7].

Modern missing data methodologies include maximum-likelihood estimation (MLE) methods such as expectation–maximisation (EM) and multiple imputation (MI), both recommended for data which is MAR [3]. MI has been shown to be robust under departures from normality, in cases of low sample size, and when the proportion of missing data is high [2]. With complete outcome variables, MI is typically less computationally expensive than MLE, and MLE tends to be problem-specific with a different model being required for each analysis [8].

Whilst many theoretical works suggest MI to be an appropriate method, it has only recently been widely applied in practice [9]. Reviews on handling missing data across different fields indicate that it is relatively rare that missing data, and how it is handled, are reported explicitly: in cost-effectiveness analysis 22 % of studies did not explicitly report missing data [10]; in education the corresponding figure is 31 % [11]; in cohort studies 16 % of studies did not report how much data was missing whilst 14 % of studies did not report how missingness was handled [12]; in epidemiology 46 % of studies were unclear about the type of missing data [13]; and in applied education and psychology 66 % of studies where the presence of missing data could be inferred did not mention missing data explicitly [6]. A review of randomised controlled trials identified 77 articles from the latter half of 2013, of which 73 reported missing data. Of these articles, 45 % performed complete case analysis, 27 % performed simple imputation (linear interpolation, worst case imputation or last observation carried forward) and only 8 % used multiple imputation [14]. Whilst MI and MLE are gaining popularity, ad-hoc techniques still appear in the applied literature, with complete case analysis remaining as the most popular approach.

Large scale survey data presents a number of challenges to imputation: a high number of variables; complexity of the data set; categorical (non-Normal) variables; categories with low observed frequency (sparsity in responses); questions which are conditional upon previous responses; and multiple multi-item scales, which are summed (either directly or weighted) during analysis. Such challenges reduce the use of sophisticated imputation techniques. As missing data in a single item of a multi-item scale leads to a missing total, the rate of missing data in scale totals can be very high. Imputing at the level of scale total whilst ignoring individual items may therefore introduce unnecessary bias. The widely-used EQ 5D-3L is one such scale, consisting of 5 items. A recent study considered imputing at item level rather than imputing scale totals [15]. When the pattern of missingness tended towards all items being missing for a respondent, little difference was seen between methods. When the pattern of missingness tended towards individual items being missing, for sample sizes of n > 100, imputing at item level was shown to be more accurate.

Another study proposed methods for handling multi-item scales at the item score level [16], and further emphasised how mean imputation or single imputation leads to bias and underestimation of standard errors. The study concludes that missing data should be handled by applying multiple imputation to the individual items. However, the size and complexity of large survey data can cause complete MI prediction models to fail to converge when the model is specified at item level, rendering the ideal method computationally infeasible.

The present study aims to develop an imputation method which addresses the challenges presented by large scale survey data, reducing the size of the prediction model whilst allowing for item level imputation. A simulation study presents a comparison of our proposed method with alternative imputation approaches, and the proposed method is illustrated further using data from a large multinational survey as a case study.

## Methods

### Multiple imputation by chained equations

Multiple imputation for a single incomplete variable works by constructing an imputation model relating the incomplete variable to other variables and drawing from the posterior predictive distribution of the missing data conditional on the observed data [1]. The approach allows for uncertainty in the missing data values by introducing variability in the imputed items.

To handle multiple incomplete variables we use multiple imputation by chained equations (MICE) which allows different variable types (continuous, nominal, ordered categorical) to be handled within the same data set [1].

In MICE, variables are initially ordered by level of missingness. Missing values are initially replaced for each variable, for example by drawing at random from the observed values of that variable. Imputation is then conducted on each variable sequentially using the observed and currently imputed values of all other variables in the imputation model. In order to stabilise, this imputation step (known as a cycle) is repeated (typically 10 times) to produce one imputed data set. The process is repeated until the desired number of imputed data sets is reached [1, 17].

### Imputation using subscale totals

Often, survey data contains responses to multiple multi-item scales. Imputing every item individually may lead to an unwieldy imputation model, which in extreme cases may fail to converge. In order to reduce the size of the imputation models yet retain item level imputation (and not discard data), we propose to impute responses to individual scale items, using the scale totals within prediction equations. In addition, when imputing responses to an item which forms part of a multiple multi-item scale, responses to other items from the scale should also be included.

As a simple example, suppose we have primary outcome measure *p,* n demographic variables *(d*_*1*_*… d*_*n*_*),* a multi-item scale S made up of 7 items *(s*_*1*_*… s*_*7*_*)*, and a multi-item scale T made up of 17 items *(t*_*1*_*… t*_*17*_*).*

The forms of the imputation models are:

- *d*_*1*_ is imputed using the observed and current imputed values of *p*, *d*_*2*_*… d*_*n*_, *s* and *t*, where *s* and *t* are the summed scale scores of *S* and *T*.
- *s*_*1*_ is imputed using the observed and current imputed values of *p, d*_*1*_*… d*_*n*_, *s*_*2*_*… s*_*7*_ and *t*.
- *s*_*2*_ is imputed using the observed and current imputed values of *p, d*_*1*_*… d*_*n*_, s_1_, *s*_*3*_*… s*_*7*_, and *t*.
- *t*_*1*_ is imputed using the observed and current imputed values of *p, d*_*1*_*… d*_*n*_, *s* and *t*_*2*_*… t*_*17*_.
- *t*_*2*_ is imputed using the observed and current imputed values of *p, d*_*1*_*… d*_*n*_, *s, t*_*1*_ and *t*_*3*_*… t*_*17*_.

with similar imputation models for *d*_*2*_*… d*_*n*_, *s*_*2*_*… s*_*7*_ and *t*_*3*_*… t*_*17*_. The proposed approach condenses information from other scales to reduce the number of predictors in each equation. Subscale totals are recalculated after each cycle of imputation.

### Categorical variables

Survey data typically contains categorical variables, which may be either nominal or ordered. Ordered categorical variables, often in the form of Likert scales, can be imputed using ordinal logistic regression, whilst nominal categorical variables may be imputed using multinomial logistic regression. Sparsity may cause non-convergence errors during multinomial logistic regression, a recognised problem [1, 18]. On occasion, this may require response categories to be collapsed prior to imputation.

### Conditional imputation

Survey design may contain some conditional questions. For example a question on experience of a specific drug will only be relevant to someone who has taken it. Within the statistical package, Stata, multiple imputation has options for conditional imputation within the -ice- routine [19]. Responses to the second part of the question are only imputed, given a certain answer to the first part of the question.

### Analysing multiply imputed data

During analysis, each of the *M* imputed data sets are analysed individually. Imputation-specific coefficients are then pooled using Rubin’s rules, to produce a single result [20]. Rubin’s rules allow the incorporation of both within imputation variance (accounting for uncertainty if the data were complete), and between imputation variance (accounting for uncertainty about the missing data) [1].

### Case study

Our data comes from an online survey, designed to investigate associations between putative predictors of adherence to antihypertensive medication, and patients’ self-reported adherence. Detailed methods of the survey and the main findings are published elsewhere [21]. Briefly, cross-sectional survey data from 2595 respondents from nine European countries (Poland, Wales, England, Hungary, Austria, Germany, Greece, the Netherlands and Belgium) was collected using the online tool SurveyMonkey^®^. The target population was adult hypertensive patients who have been prescribed antihypertensive medication.

**Fig. 1:**
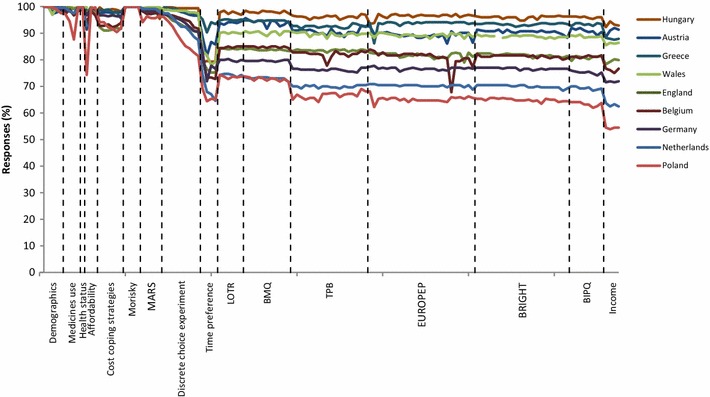
Responses rate by question and country. Questions not-applicable due differences in healthcare systems appear as breaks in the *plot*. *MARS* Medication Adherence Rating Scale, *LOTR* Life Orientation Test Revised, *BMQ* Beliefs about Medicines Questionnaire, *TPB* Theory of Planned Behaviour, *EUROPEP* European Task Force on Patient Evaluations of General Practice, *BRIGHT* Building Research Initiative Group Illness Management and Adherence in Transplantation, *BIPQ* Brief Illness Perception Questionnaire Reprinted from Value in Health, 18(2), Morrison VL, Holmes EAF, Parveen S, Plumpton CO, Clyne W, De Geest S, Dobbels F, Vrijens B, Kardas P, Hughes DA, Predictors of Self-Reported Adherence to Antihypertensive Medicines: A Multinational, Cross-Sectional Survey, 206–216, Copyright (2015), with permission from Elsevier [21]

The survey comprised 13 validated measures from health psychology and behavioural economics, alongside demographic questions, resulting in a total of 135 questions. Within the health psychology sections, responses to several questions were summed to form subscale totals, as per validated approaches to analysing these measures. There are a total of 14 such subscales within the survey. Due to the length of the survey, a level of missing data was to be expected, with respondents dropping out part way through or skipping one or more question. We ensured no missingness in the primary outcome measure, the Morisky measure of adherence [22], by enabling ‘forced answer’ settings within SurveyMonkey. Figure 1 presents the percentage of complete responses by question, and in the order the questions were asked. A dip is seen at the open ended time preference measure, which may be perceived as cognitively challenging [23]. The sensitivity of information requested on income explains the final dip in the plot. Missing data was assumed to be MAR. We consider the impact of possible departures from MAR in the discussion.

We chose to impute each country-specific data set separately, as associations between variables were expected to differ between countries.

A complete MI prediction equation results in 134 predictors for each incomplete variable. Some categorical variables had categories with low observed frequency which presented additional challenges. These were handled by collapsing response categories. Taking education as an example, in Greece, 52.3 % received primary education as a highest educational attainment, 29.0 % secondary education and 18.7 % higher education. For England the corresponding figures were 0.3, 33.7 and 65.3 % respectively. We collapsed the lower two categories, conducting the final analysis on ‘up to secondary education’ and ‘higher education’. Collapsing of categories was applied to all data sets, and was maintained for analysis.

Within the income section of the survey, questions had an ‘opt out’ response if respondents were unwilling to provide the information. Additional file 1: Table S1 summarises these responses across the nine countries. Questions in this section took an ordered categorical format, which we were keen to preserve (rather than impute as nominal variables). This was achieved by generating two separate variables for each income item. An initial binary variable reflected whether the respondent was willing to provide a response. An ordinal variable then reflected the response, conditional on the respondent being willing to provide the information.

The number of imputations, *M*, was chosen based upon analysis of the Polish data set, which was received 3 months prior to data from other countries. For this data set 26 % of response values were missing, so the number of imputations was set at *M* = *25*, closely reflecting the suggestion of one imputation per percent missing data [1]. For subsequent country-level data sets the amount of missing data was in fact lower, 5–22 %, but *M* = *25* was maintained for consistency.

Initially a full imputation model was attempted for this data, but failed to converge for all imputations. Applying our proposed approach, model size depended on the number of items within each subscale (range, 56–72). Each prediction equation included the demographic variables and the primary outcome measure. MI was conducted using the -ice- routine in Stata 10 [19, 24, 25]. Categorical variables were handled using -mlogit- and -ologit- [25]. Subscale totals were calculated following each cycle of the imputation using the passive option of the -ice- routine. The final imputation methods and models used to impute different parts of the survey are summarised in Table 1, with an extract of Stata code provided in Additional file 2: Appendix S1.

**Table 1:** Missing data by country, and how it was handled in imputation models

Variables	% incomplete (for scale variables, % of missing responses to scale items, not scale totals)									Imputed as	Used in imputation models as
	Austria	Belgium	England	Germany	Greece	Hungary	Netherlands	Poland	Wales		
Total N	323	180	323	274	289	323	237	323	323		
Gender	0	0	0	0	0	0	0	0	0	1 binary item	Single item
Age	0	0	0	0	0	0	0	0	0	1 continuous item	Single item
Education	3	0	1	0	2	1	0	0	0	1 categorical item	Single item
Marital status	2	0	0	1	1	1	0	1	0	1 binary item	Single item
Employment	2	0	0	1	1	1	0	2	0	1 binary item	Single item
Number of											
Medical conditions	1	3	0	1	1	0	0	3	0	1 continuous item	Single item
Medicines	1	1	0	2	1	1	2	6	1	1 continuous item	Single item
Tablets	3	3	1	3	1	1	3	12	1	1 continuous item	Single item
Items prescribed	9	6	3	3	1	8	7	26	6	1 continuous item	Single item
Dosage frequency	1	2	0	0	1	0	1	0	0	1 ordered categorical item	Single item
Morisky adherence	0	0	0	0	0	0	0	0	0	4 binary items	Binary item
Medication adherence rating scale	2	3	1	2	1	2	1	4	0	5 ordinal items	Scale
Prescription payment	1	2	0	1	1	1	*	1	*	1 categorical item	Single item
Affordability problem	1	3	2	1	1	3	1	0	*	1 binary item	Single item
Cost coping strategies	2	7	8	3	2	1	*	8	*	6 ordered categorical items	6 single items
Income											
Source	11	23	22	28	12	8	37	46	15	1 categorical item	Single item
Perception	8	25	20	28	12	7	37	46	14	2 items: ordered categorical item conditional on binary item	2 items
Ease of borrowing	9	23	20	28	12	7	38	48	14	2 items: ordered categorical item conditional on binary item	2 items
Total	9	24	21	28	12	6	38	46	14	2 items: ordered categorical item conditional on binary item	2 items
Health status	1	1	0	0	0	0	1	0	0	1 ordered categorical item	Single item
Practitioner											
Type	7	16	17	23	6	7	29	32	10	1 categorical item	Single item
Gender	10	17	18	22	14	6	29	38	12	1 binary item	Single item
Satisfaction with											
Practitioner	11	18	18	28	2	3	30	35	7	17 ordered categorical items	Scale
Practice	11	23	18	23	6	3	30	34	11	6 ordered categorical items	Scale
Optimism	6	15	16	20	5	2	26	26	10	6 ordered categorical items	Scale
Illness perception questionnaire: analysed as 8 individual items	9	19	19	25	7	4	31	37	11	8 ordered categorical items	Scale
Necessities	6	15	16	20	5	2	27	27	10	5 ordered categorical items	Scale
Medicine concerns	6	15	16	20	5	2	27	27	10	6 ordered categorical items	Scale
Attitude	9	17	16	24	7	4	30	35	10	7 ordered categorical items	Scale
Barriers (theory of planned behaviour)	10	17	16	23	7	3	30	33	11	1 ordered categorical item	Single item
Facilitators	11	18	16	24	8	5	30	34	11	3 ordered categorical items	Scale
Intention	10	18	17	24	8	4	30	33	10	2 ordered categorical items	Scale
Self efficacy	7	1	16	23	6	3	30	31	10	2 ordered categorical items	Scale
Normative beliefs	10	20	17	24	7	4	30	33	11	3 ordered categorical items	Scale
Barriers	21	22	22	38	18	6	35	35	9	15 ordered categorical items	Scale
Social support	10	19	19	23	20	4	31	43	11	7 ordered categorical items	Scale
Time preference	14	25	23	23	7	21	32	34	19	4 ordered categorical items	4 single items
Discrete choice experiment: not included in analysis	2	5	7	6	2	1	7	12	2	9 binary items	9 single items

Unless stated, variables correspond to a single predictor during analysis. Due to differences in healthcare and prescription systems between countries, not all questions applied to each country. Additionally, in Wales, one question from the barriers scale was not applicable, thus this scale has only 14 items. Whilst illness perception questions were imputed as scale items, they were analysed individually

* Variables not analysed due to differences in prescription policies

### Data analysis

Primary analysis was to be conducted by country, and the survey was powered as such. The primary analysis was a logistic regression with Morisky score as outcome, aiming to identify predictors of non-adherence to medication. There were deemed to be too many predictors to enter into the model, N = 42 (Table 1), therefore an initial variable selection step was employed. For the regression results presented here, we have used the same pragmatic variable selection as in the main analysis [21]: continuous variables were selected using univariate tests, pooled using Rubin’s rules; categorical variables were selected using Chi squared tests and ANOVA on complete case data; and variables relating to numbers of medicines were selected using t-tests controlling for age on complete case data. Variables showing univariate significance with the outcome measure were entered into the regression model.

We also compared variable selection using complete case data with variable selection procedure using Rubin’s rules in the pooled MI data, using unadjusted or age-adjusted analyses as described above [20, 26].

### Simulation

We devised a simulation study to impartially assess the performance of the new method against some alternatives in a realistic setting—based on the case study.

We invoked a simpler set up than the case study, to allow comparison of the proposed strategy with a full imputation model, which is not possible on the full data set. The variables included were *Morisky score* (fully observed), *age* in years (fully observed), *attitude* (partially observed, the sum of seven items, scored as integers from 1 to 5) and *practitioner satisfaction* (partially observed, the sum of 17 items, also integers from 1 to 5). We estimated four quantities: the means of *attitude* and *practitioner satisfaction*, and their coefficients in a logistic regression of Morisky score on *age*, *attitude* and *practitioner satisfaction*.

### Simulation procedure

The simulation procedure was as follows for 1000 replications:

- 323 observations of the 26 variables (Morisky score, age, 7 items of attitude, 17 items of practitioner satisfaction) were simulated from a multivariate normal distribution based on the observed vectors of means and standard deviations and the observed correlation matrix.
- Morisky score was rounded to the nearest of 0 or 1. Items making up *attitude* and *practitioner satisfaction* were rounded to take values of 1–5.
- Missing values were introduced for items of the *attitude* and *practitioner satisfaction* scales. The probability of missing data depended on *Moriskyscore* and *age*, based on the real dataset (MAR). Each observation was assigned to one of three categories: all items observed, some items observed, or no items observed. Three scenarios are simulated:
  - Base case: 35 % had all items missing for a scale; 8 % had one or two items missing.
  - More incomplete observations with partial data: 18 % had all items missing for a scale; 25 % had one or two items missing.
  - Fewer observations with complete data: 55 % had all items missing for a scale; 15 % had just one or two missing.

For each simulated dataset six methods were considered for dealing with the missing data, presented in Table 2. Ten imputations were used for all MI-based approaches.

**Table 2:** Summary of methods compared in simulation

Method description	Assumptions	Comments
1. Exclude observations with any missing data from the analysis	Missing values are independent of Morisky score given the other variables	Complete case analysis
2. For partially observed scales, sum the observed values, weighted by (1/proportion of items observed). Exclude observations with completely missing scales	Partially observed items are MAR given other items in the scale and completely missing scales are MCAR	Effectively single imputation as the mean of observed items within a scale
3. For partially observed scales, set the score to missing. Multiply impute the scale sums from a multivariate normal model with Morisky score and age as covariates	Missingness is MAR, and this process is the same for missing scales or missing items within scales	Wasteful of observed data
4. For partially observed scales, sum the observed values, weighted by (1/proportion of items observed). For completely missing scales, multiply impute the scale sums from a multivariate normal model with Morisky score and age as covariates	Completely missing scales are MAR	Uses single imputation in the same way as approach 2
5. Multiply impute missing items based on the total of the other scale, and the other items within the scale for the item being imputed (with Morisky score and age as covariates). This requires the use of chained equations with linear regression imputation rather than a multivariate normal model	Missing at random for both variables, but that the regression for one item on the other scale items is the same as the regression on the other scale total	Proposed adaptation
6. Multiply impute all items using all other items via a multivariate normal model, including Morisky score and age as covariates	Multivariate normality	It is in some senses the benchmark

### Outcomes

For each parameter of interest we summarise percent bias (compared to analysis of complete data), coverage, and efficiency (through the empirical standard error, expressed by comparison to method 1) over the 1000 replications for that scenario. Estimates are accompanied by Monte-Carlo 95 % confidence intervals.

## Results

### Case study

**Fig. 2:**
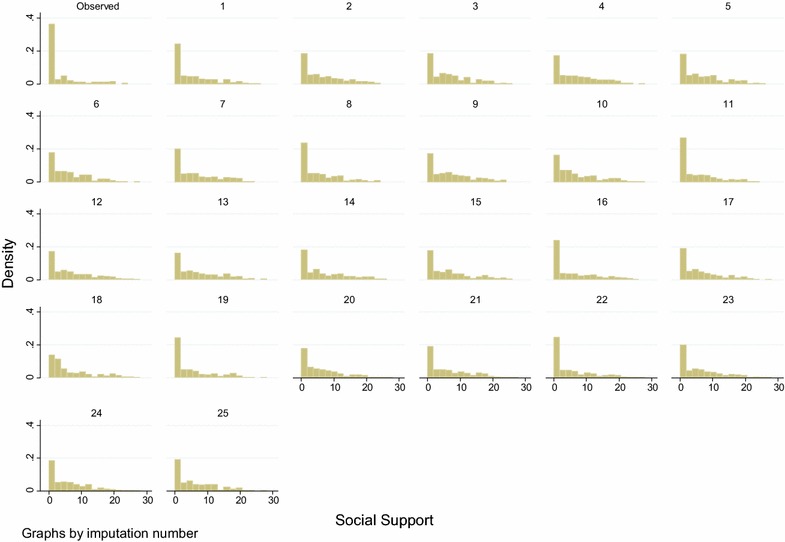
Social-support scale for Poland, 70 % totals and 43 % individual items missing

**Fig. 3:**
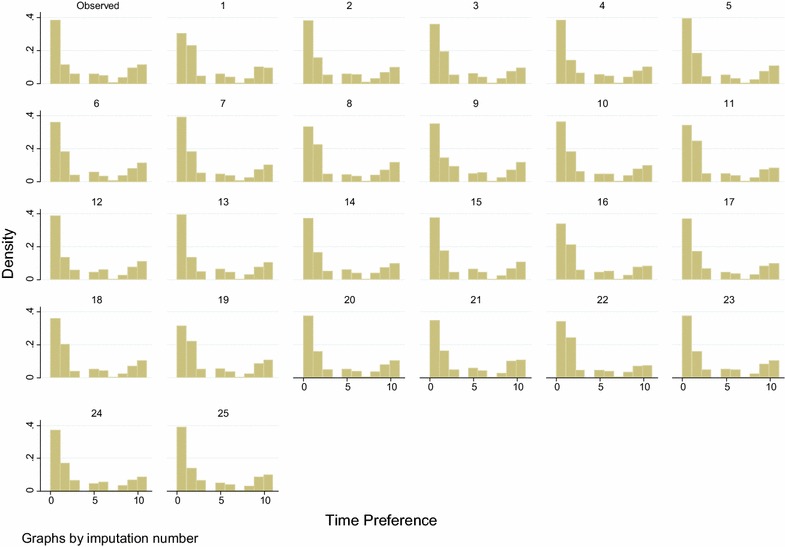
Individual item, time preference variable 2, for Poland, 36 % missing

To compare the performance and fit of the MI models, we plot complete case data versus imputed data, overall and by imputation. Figures 2 and 3 illustrate such comparisons for the individual item and scale total which displayed the highest proportion of missing data. These are one of the time preference variables (36 % missing data), and the support scale (70 % of scale totals missing, 43 % individual items missing), both from the Polish data set. On inspection, in both cases, the imputed data is similar but not identical to the complete case data.

**Fig. 4:**
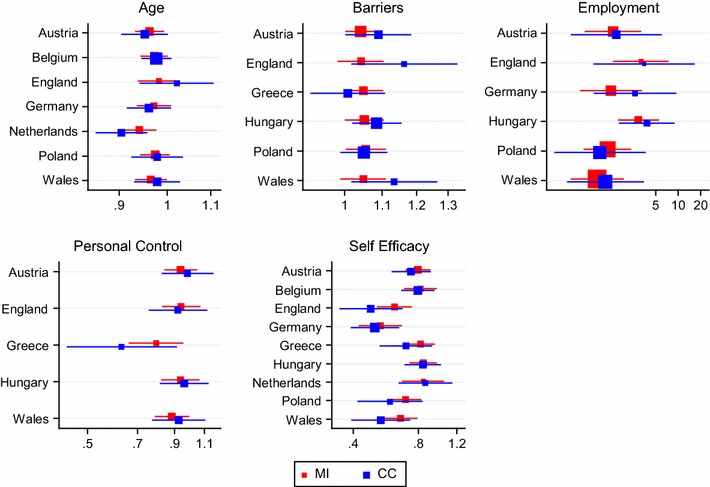
*Forest plots* illustrating odds ratios from the logistic regression

For those variables which were entered into the regression model in five or more countries, the regression results are illustrated as odds ratios with 95 % confidence intervals in Fig. 4. Differences in the significance of results are observed between data analysed using MI and CC for age, barriers and personal control in Austria, barriers and self-efficacy in England, barriers and employment in Poland and age in Wales. The majority of differences (except barriers in Austria and Poland) are attributable to narrower confidence intervals in the MI analysis, thus illustrating the higher power and efficiency which the MI approach offers. Whilst differences in the standard errors alter the significance of the results, there are no substantial differences in the point estimates of the β-coefficients.

Table 3 presents the proportional reduction in standard error for MI compared to CC analysis, summarised for all variables entered in the country level regression analyses. From the table, it can be seen that on average, standard error is reduced by 39 % when an MI approach is adopted over CC analysis. Standard errors are smaller for MI than CC analysis for all variables other than illness coherence in Belgium (where there was no change in standard error between MI and CC). To ensure that reduction in standard error was not biased by variable selection method, reduction was also compared for variables selected using CC and MI approaches. For variables selected using the CC method, mean standard error reduction was 45 % (95 % CI: 12, 78 %; range 15–99.9 %). For variables selected using the MI method, mean standard error reduction was 34 % (95 % CI: 11, 58 %; range 2–57 %).

**Table 3:** Summary of proportional decrease in standard error, between complete case and multiple imputation analyses

	Mean (%)	Min (%)	Max (%)	Median (%)	Standard deviation (%)
Overall	39	0	100	38	19
Austria	38	22	55	38	8
Belgium	5	0	13	4	5
England	58	33	100	45	27
Germany	21	12	26	22	5
Greece	50	43	58	50	4
Hungary	23	14	27	23	4
Netherlands	29	24	36	29	4
Poland	41	14	59	42	12
Wales	36	28	47	37	6

For the univariate variable selection, disparity between which variables were selected using either the MI or CC approach is summarised in Table 4. Chi squared tests indicate that the disparity was significant, (χ^2^ = 250, p < 0.001), with agreement (sum of the main diagonal) achieved for only 92.5 % of variables. Lower agreement is observed in the variables with more missing data: at <20 % missing data agreement was 94 %, compared to 88 % when missing data was > 20%.

**Table 4:** Disparity in variable selection between CC and MI, over 42 variables in 9 countries

		Complete case method		
		Included n (%)	Excluded n (%)	Total
Multiple imputation	Included n (%)	86 (23)	3 (1)	89
	Excluded n (%)	25 (7)	259 (69)	284
	Total	111	262	373

χ^2^= 250, p < 0.001

### Simulation

**Fig. 5:**
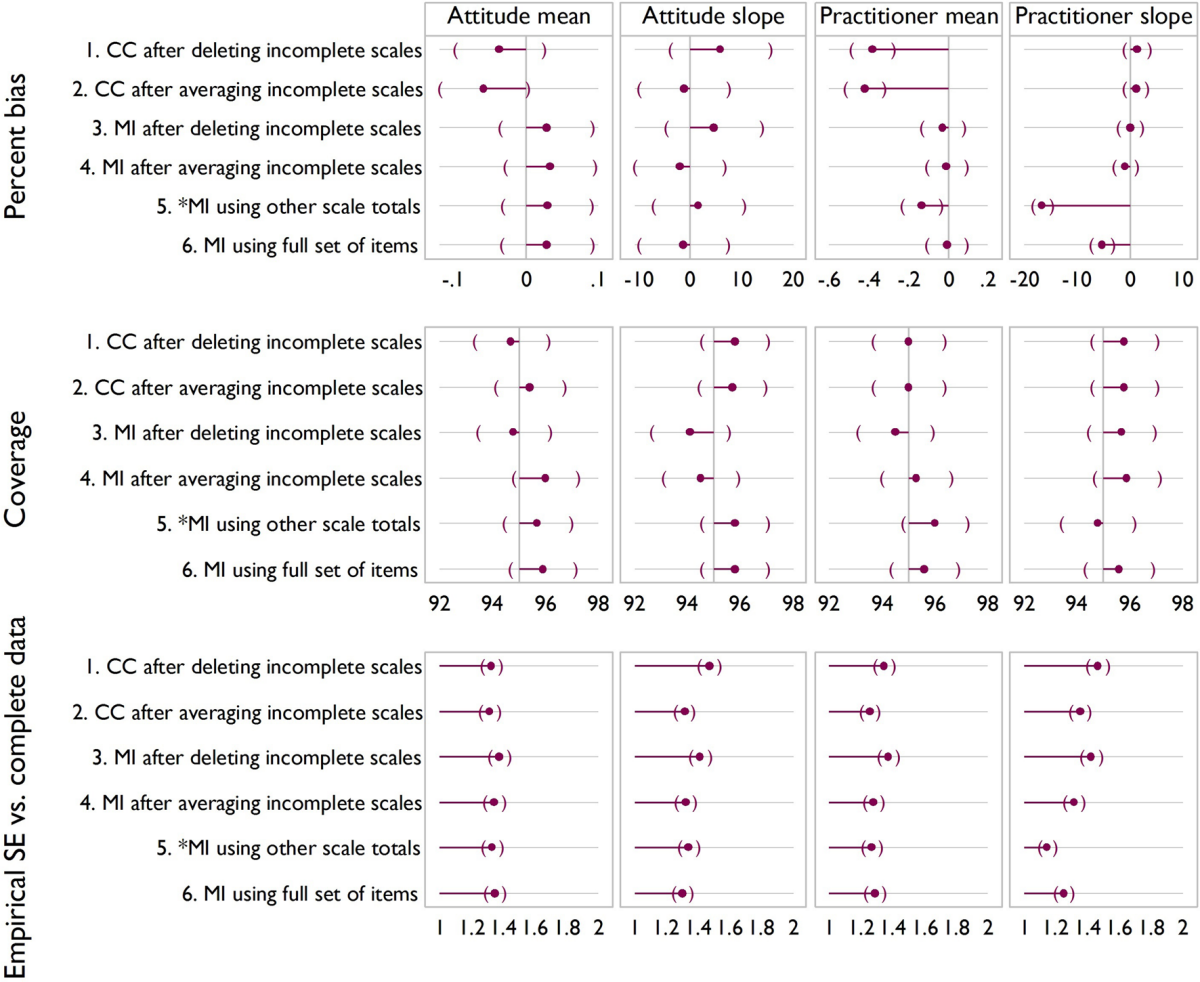
Simulation results for the three scenarios. *Brackets* indicate confidence intervals. Base case: 35 % had all items missing for a scale; 8 % had one or two items missing

**Fig. 6:**
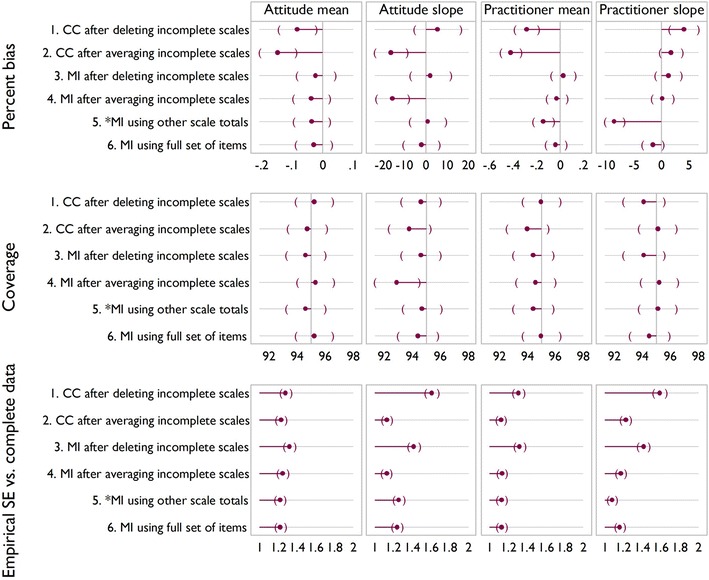
Simulation results for the three scenarios. *Brackets* indicate confidence intervals. More incomplete observations with partial data: 18 % had all items missing for a scale; 25 % had one or two items missing

**Fig. 7:**
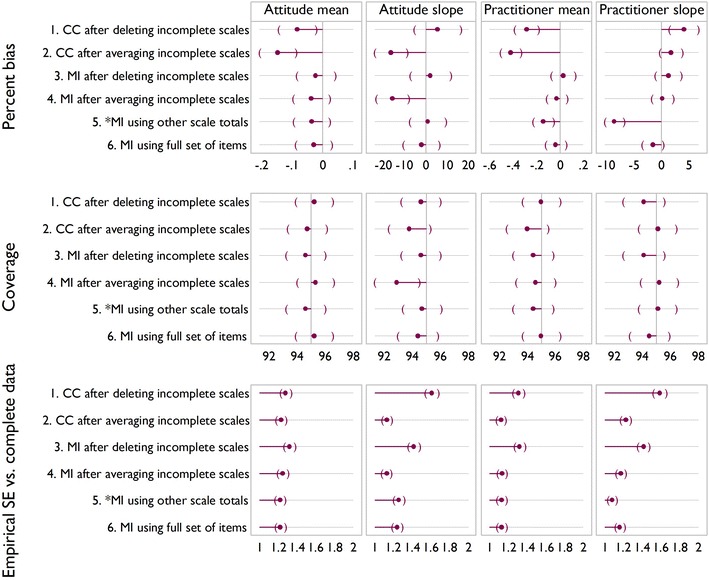
Simulation results for the three scenarios. *Brackets* indicate confidence intervals. Fewer observations with complete data: 55 % had all items missing for a scale; 15 % had just one or two missing

Figures 5, 6 and 7 show the results of the simulation for the three scenarios. In the base case Fig. 5, significant downward bias is seen for the mean of practitioner satisfaction, for methods 1, 2 and 5, with methods 5 and 6 showing significant bias on the slope. In terms of coverage, there are no significant differences between methods. Empirical standard error also shows little variability between methods, except that it is lower for method 5 on slope for practitioner satisfaction. This reflects the downward bias.

Increasing the number of incomplete observations with partial data, as in scenario 2 (Fig. 6) or increasing the number of incomplete observations (Fig. 7) indicate a similar story. Methods 1 and 2 show an increase in bias compared to the base case, with method 4 showing significant downward bias and reduced coverage for TPB slope in both scenarios. In both scenarios the empirical standard error appears lower than for the base case, reflecting downward bias.

Overall, method 6 is seen to be the best, broadly exhibiting the least bias and the most efficiency, and we regard it as a benchmark. This method is not always feasible however, for example in the case study described above. Method 1 often displays a large amount of bias, and like method 3, is inefficient and wasteful of observed data. Method 2 indicates bias in all bar the base case, and may artificially reduce variability due to being effectively single imputation.

It appears therefore from the simulations and assumptions that in terms of bias, coverage and empirical standard error that method 4 or 5 would be best in cases where method 6 is not feasible. At this point it is unclear which of the two methods is most appropriate, method 4, similar to method 2 is akin to a single imputation, and for method 5 whilst the assumptions seem more appropriate, the simulation evidence suggests it can introduce bias when these are violated.

## Discussion

Our proposed method for handling multiple multi-item scales allows imputation of individual items by using scale totals within the imputation models, such that given primary outcome *p*, scale *T* and demographics *d*_1_ … *d*_n_, item *s*_1_ from scale *S*, is imputed using *p*, *d*_1_ … *d*_n_, *s*_2_ … *s*_n_ and summary score *t*. The use of summed scale scores within the predictor equations reduces both the number of predictors in each equation and the sparsity in the data set. This approach facilitates the efficient use of MI in large survey data sets with multiple multi-item scales. Using MICE allows preservation of the structure of the data, in terms of point estimates and variance or variables, and covariance. Should the approach presented here still result in overly complex prediction equations, a further simplification would be to replace *s*_2_ … *s*_n_ in the prediction equation by their sum or average.

For subscales of the health psychology measures, rather than to impute every individual item, one simplification of our method would be to impute only the totals of the subscales. For our data this would reduce the size of the model from 134 to 56 predictors per variable. A disadvantage of this approach, however, is that it would restrict analysis to summed scales, leaving no scope for exploring individual items.

Forming scale totals prior to imputation, and then imputing missing totals is a further simplification, but comes with an additional disadvantage: for those respondents who have completed some, but not all, of the items in a subscale, those responses are discarded, or imputed by an ad-hoc method such as using the mean of observed items. Taking as an example the 17-item practitioner satisfaction scale in the Austrian data set, 262 respondents (from 323) completed all items. The response rate to individual items ranged from 278 to 292 responses, and the above approach would discard a total of 437 responses, collected from 31 respondents, from this scale.

Our simulation study compares these alternatives with a benchmark ‘complete’ MI analysis, and complete case alternatives. Results from the simulation indicate that simplification by averaging incomplete scales and our proposed method perform comparably, with the simplification reducing model complexity, compromised with a slight loss of efficiency. Complete case methods were seen to perform poorly, with an increase in bias, particularly when the amount of missingness was increased. This result is consistent with a previous study on multi-item imputation, where mean imputation and single imputation were shown to have larger bias and worse coverage than item level multiple imputation, and complete case analysis was shown to overestimate standard error and reduce power [16].

Certain limitations are acknowledged. Typically, analysis with MI relies on an assumption of data being MAR. This assumption cannot be proven, but for large well-conducted surveys, the assumption of MAR is often considered a reasonable starting point for statistical analysis. Rubin et al. conclude that whilst assuming MAR may be inadequate if there are high levels of missingness or insufficient relevant covariates, “the evidence seems to be that at least in some carefully designed survey contexts, modelling observed data under MAR can provide acceptable answers” [27].

A further limitation of the proposed method for handling multiple multi-item scales is the assumption that items from different scales are only correlated via the scale totals. The majority of scales and measures used in this survey are validated, and were tested during analysis for internal consistency. Additionally, as the survey was structured such that each measure was presented in its entirety, independently, this a plausible assumption for our data.

During data analysis, significant univariate differences were observed between CC and MI which indicate that performing variable selection on different data sets would result in the entry of different variables into the regression model. In comparison with CC, using the same variables for both approaches, regression results from MI show standard error to be reduced by an average of 39 %, with no cases where standard error increases, thus resulting in more precise conclusions.

Data collection will almost always result in missing data. It is the duty of researchers and analysts to firstly minimise the extent of missing data by ensuring appropriate methods for enhancing data capture are implemented, but also to handle missingness in a way best suited to the data and research question. Poor handling and reporting of missing data may result in misleading conclusions and are one of the main reasons for publication rejections [28, 29]. With the advent of MI routines in SPSS, R, SAS and Stata, MI is now readily accessible to analysts as a robust method for handling missing data, which can be applied in a number of contexts [4].

Alongside our proposed adaptation for imputing multiple multi-item scales, ordinal regression and conditional imputation are also powerful tools which, in this study, have allowed us to address the challenges presented by large scale survey data. Our proposed adaptation makes MI practical for large scale survey data, where a full prediction model may be infeasible, and we have shown that the use of MI in this way makes better use of available data and can yield substantively different results from simpler techniques.

## Additional files

10.1186/s13104-016-1853-5 Stata code.
10.1186/s13104-016-1853-5 Responses to income questions.
