# Supplementary material for: Multiple imputation of multiple multi-item scales when a full imputation model is infeasible
Source: BMC Res Notes. 2016 Jan 26;9:45. doi: 10.1186/s13104-016-1853-5 (PMC4727289; doi:10.1186/s13104-016-1853-5)
Supplement: Supplementary file 1 — 10.1186/s13104-016-1853-5 Stata code. [file 13104_2016_1853_MOESM1_ESM.docx]

WebAppendix 1

*Stata code*

/* Run MICE */

#delimit ;

ice /* Specify variables to be imputed, with o. for ordered categorical */

D_Sex D_Age o.MARS1 o.MARS2 o.MARS3 o.MARS4 o.MARS5 o.LOTR1

o.LOTR3 o.LOTR4 o.LOTR7 o.LOTR9 o.LOTR10 income2_willing o.income2_ordinal,

eq( /* Specify imputation models */

D_Age: D_Sex Morisky MARS LOTR income2*,

D_Sex: D_Age Morisky MARS LOTR income2*,

MARS1: D_* Morisky MARS2 MARS3 MARS4 MARS5 LOTR income2*,

MARS2: D_* Morisky MARS1 MARS3 MARS4 MARS5 LOTR income2*,

MARS3: D_* Morisky MARS1 MARS2 MARS4 MARS5 LOTR income2*,

MARS4: D_* Morisky MARS1 MARS2 MARS3 MARS5 LOTR income2*,

MARS5: D_* Morisky MARS1 MARS2 MARS3 MARS4 LOTR income2*,

LOTR1: D_* Morisky MARS LOTR2 LOTR3 LOTR4 LOTR5 LOTR6 income2*,

LOTR2: D_* Morisky MARS LOTR1 LOTR3 LOTR4 LOTR5 LOTR6 income2*,

LOTR3: D_* Morisky MARS LOTR1 LOTR2 LOTR4 LOTR5 LOTR6 income2*,

LOTR4: D_* Morisky MARS LOTR1 LOTR2 LOTR3 LOTR5 LOTR6 income2*,

LOTR5: D_* Morisky MARS LOTR1 LOTR2 LOTR3 LOTR4 LOTR6 income2*,

LOTR6: D_* Morisky MARS LOTR1 LOTR2 LOTR3 LOTR4 LOTR5 income2*,

income2_ willing: D_* Morisky MARS LOTR, income2_ordinal: D_* Morisky MARS LOTR) cond(income2_ordinal: income2_willing==1)

passive( /* Scale totals are computed within each MICE cycle */

MARS: MARS1+MARS2+MARS3+MARS4+MARS5 \

LOTR: LOTR1+LOTR3+LOTR4+LOTR7+LOTR9+LOTR10)

saving(imputed_extract) m(25);
