# Supplementary material for: Multiple imputation of multiple multi-item scales when a full imputation model is infeasible
Source: BMC Res Notes. 2016 Jan 26;9:45. doi: 10.1186/s13104-016-1853-5 (PMC4727289; doi:10.1186/s13104-016-1853-5)
Supplement: Supplementary file 2 — 10.1186/s13104-016-1853-5 Responses to income questions. [file 13104_2016_1853_MOESM2_ESM.docx]

WebTable 1

Responses to Income questions

|  |  | Income source | Total income | Income perception | Ease of borrowing |
| --- | --- | --- | --- | --- | --- |
| Poland  N=323 | Not willing to provide (%) | 1 | 9 | 2 | 4 |
|  | Missing (%) | 46 | 46 | 46 | 46 |
| Wales  N=323 | Not willing to provide (%) | 4 | 10 | 4 | 9 |
|  | Missing (%) | 15 | 14 | 14 | 14 |
| England  N=323 | Not willing to provide (%) | 3 |  | 4 | 7 |
|  | Missing (%) | 22 | 21 | 20 | 20 |
| Hungary  N=323 | Not willing to provide (%) | 18 | 24 | 20 | 32 |
|  | Missing (%) | 8 | 6 | 7 | 7 |
| Austria  N=323 | Not willing to provide (%) | 9 | 12 | 11 | 15 |
|  | Missing (%) | 11 | 9 | 8 | 9 |
| Belgium  N=180 | Not willing to provide (%) | 3 | 12 | 5 | 12 |
|  | Missing (%) | 23 | 24 | 25 | 23 |
| Germany  N=274 | Not willing to provide (%) | 2 | 8 | 5 | 8 |
|  | Missing (%) | 28 | 28 | 28 | 28 |
| Greece  N=289 | Not willing to provide (%) | 2 | 9 | 3 | 6 |
|  | Missing (%) | 12 | 12 | 12 | 12 |
| Netherlands  N=237 | Not willing to provide (%) | 4 | 10 | 6 | 9 |
|  | Missing (%) | 36 | 38 | 37 | 38 |
